# Transcriptomic Response of *Fusarium verticillioides* to Variably Inhibitory Environmental Isolates of *Streptomyces*

**DOI:** 10.3389/ffunb.2022.894590

**Published:** 2022-07-28

**Authors:** Timothy R. Satterlee, Felicia N. Williams, Marina Nadal, Anthony E. Glenn, Lily W. Lofton, Mary V. Duke, Brian E. Scheffler, Scott E. Gold

**Affiliations:** ^1^ United States Department of Agriculture (USDA), Agricultural Research Service (ARS), Toxicology and Mycotoxin Research Unit, United States (US) National Poultry Research Center, Athens, GA, United States; ^2^ United States Department of Agriculture (USDA), Agricultural Research Service (ARS), Genomics and Bioinformatics Research Unit, Stoneville, MS, United States

**Keywords:** *Fusarium verticillioides*, *Streptomyces*, mycotoxins, microbial interactions, transcriptome, fungal beta-lactamase

## Abstract

*Fusarium verticillioides* is a mycotoxigenic fungus that is a threat to food and feed safety due to its common infection of maize, a global staple crop. A proposed strategy to combat this threat is the use of biological control bacteria that can inhibit the fungus and reduce mycotoxin contamination. In this study, the effect of multiple environmental isolates of *Streptomyces* on *F. verticillioides* was examined *via* transcriptome analysis. The *Streptomyces* strains ranged from inducing no visible response to dramatic growth inhibition. Transcriptionally, *F. verticillioides* responded proportionally to strain inhibition with either little to no transcript changes to thousands of genes being differentially expressed. Expression changes in multiple *F. verticillioides* putative secondary metabolite gene clusters was observed. Interestingly, genes involved in the fusaric acid gene cluster were suppressed by inhibitory strains of *Streptomyces.* A *F. verticillioides* beta-lactamase encoding gene (FVEG_13172) was found to be highly induced by specific inhibitory *Streptomyces* strains and its deletion increased visible response to those strains. This study demonstrates that *F. verticillioides* does not have an all or nothing response to bacteria it encounters but rather a measured response that is strain specific and proportional to the strength of inhibition.

## Introduction

As global temperatures rise with greenhouse gas levels, important staple crops are becoming more susceptible to fungal infection and mycotoxin contamination (Vaughan et al., 2014; Vaughan et al., 2016, Garcia-Cela et al., 2021, Camardo Leggieri et al., 2019, Rosa Junior et al., 2019). According to a recent global survey, mycotoxins produced by *Fusarium* spp., including fumonisins, are the most common in grain worldwide, with greater prevalence than secondary metabolites produced by *Aspergillus* or other genera (BIOMIN World Survey, 2021). *Fusarium verticillioides* is a primary producer of the mycotoxin fumonisin B1 which is associated with birth defects in humans, growth stunting as well as esophageal cancer (Chen et al., 2018). While direct ingestion of fumonisin by humans is a major concern in developing nations strongly dependent on corn, grain contaminated with fumonisins are commonly ingested by farm animals worldwide. In horses and swine, consumption of fumonisin can cause leukoencephalomalacia and pulmonary edema, respectively (Marasas et al., 1988, Ross et al., 1990). In particular for poultry, fumonisin and other *Fusarium* mycotoxins contaminate feed and can lead to lower egg quality, induce hepatic oxidative stress, and predispose the birds to necrotic enteritis (Antonissen et al., 2015, Dazuk et al., 2020; Sousa et al., 2020) potentially leading to additional food safety bacterial load (Shanmugasundaram et al., 2013; Shanmugasundaram et al., 2020).

To control mycotoxigenic fungi, like *F. verticillioides*, the use of biocontrol agents capable of inhibiting the growth of the pathogen or its ability to produce harmful metabolites is being explored. *Trichoderma* is a well-known genus of fungal biocontrol agents targeting *Fusarium* species (Larran et al., 2020; Tian et al., 2020). Compounds made by lactic acid bacteria have also shown promise to inhibit *F. verticillioides* and *Aspergillus flavus* (Nazareth et al., 2019). The *Bacillus mojavensis* strain RRC101 lipopeptide, fengycin, caused colony growth inhibition and dramatic microscopic *F. verticillioides* hyphal swellings that rapidly rupture (Blacutt et al., 2016).

*Streptomyces* species are actinobacteria of interest for biological control of fungal plant pathogens. Multiple studies have evaluated the effectiveness of specific strains to inhibit an array of *Fusarium* spp. An endophytic strain of *Streptomyces* from *Pinus dabeshanensis*, known as WP-1, was shown to prevent conidial germination and inhibit the mycelial growth of *Fusarium oxysporum* (Peng et al., 2020). *Streptomyces pratensis* S10, a hyperparasite of *Fusarium graminearum*, was able to prevent wheat infection and suppress activation of the biosynthetic genes involved in deoxynivalenol production (Zhang et al., 2020). Another example is the use of *Streptomyces* to combat *Fusarium* wilt of bananas (Jing et al., 2020). An isolate known as *Streptomyces* sp AV-05 was able to inhibit fumonisin production in *F. verticillioides* (Strub et al., 2021).

*Streptomyces* and *Fusarium* are known to directly compete for overlapping nutritional niches (Essarioui et al., 2017, Essarioui et al., 2019). This competition contributes to a coevolutionary arms race with each species producing metabolites to impede the other (Essarioui et al., 2017, Essarioui et al., 2019). Interestingly, inhibition can be more pronounced if the microbes are from sympatric locations, but inhibitory interactions may still be evident even if microbes are from allopatric locations (Essarioui et al., 2020).

In this study, we focused on correllation of temporal transcriptomic responses of *F. verticillioides* strain FRC M-3125 (FGSC 7600; the genome reference strain) to four *Streptomyces* environmental isolates of potential biological control value. The four strains selected were from nine initially screened and represent the range of visible inhibitory impacts on *F. verticillioides* growth. After initial independent growth, M3125 was exposed in a time course to the diffusible culture metabolites of the four *Streptomyces* strains. The results document the unique responses that *F. verticillioides* demonstrated to specific inhibitory strains of *Streptomyces*. Key responses included differential expression of various secondary metabolite biosythetic gene clusters and common stress response elements. Finally, deletion of an induced beta-lactamase encoding gene resulted in increased pigmentation response to these inhibitory strains.

## Materials and Methods

### Strains and Culture Conditions

All strains used in this study are listed in **Table S1**. Strains were cultured on solid CZY medium (0.5% w/v yeast extract, 50g of Czapek Dox agar per liter) and incubated at 28°C in the dark unless otherwise stated. For dual culture assays, *Streptomyces* isolates were initially inoculated into liquid CZY (0.5% w/v yeast extract, 35g of Czapek Dox broth per liter) with shaking at 250 rpm for 48 h to produce a dense culture. Cells were collected by centrifugation and rinsed with and resuspended in sterile deionized water for use as inoculum. For *F. verticillioides* inoculum, spores were collected from three-day old PDA cultures in sterile water, and then rinsed to remove excess media. For inhibition assays, *Streptomyces* strains were inoculated in a straight line 25 mm from the plate center and grown for 48 h on solid CZY. Then *F. verticillioides* spores were point inoculated with 10^4^ spores in 5 µl at plate center and incubated five more days.

For the RNA-Seq experiment, the inoculum was prepared as above, and the bacteria were applied as a circular ring of inoculum to 25 ml of CZY agar in 100 mm diameter plates. This ring was created by using the rim of a small (60 mm diameter) Petri plate lid that was dipped into a thick suspension of *Streptomyces* cells and then stamped onto the CZY agar in the larger plates (**Supplementary Figure 1**). The bacterial ring culture was grown for 48 h. At the same time, *F. verticillioides* was independently grown on 47 mm cellulose membranes filters (Fisher Scientific Cat No 09-719-555) atop 60 mm CZY plates at 28°C in the dark. After the initial 2-day incubation, each *F. verticillioides* colonized membrane was placed in the center of a bacterial ring culture as seen in **Supplementary Figure 1**, allowing for no direct physical contact between species. Cultures were grown together for up to 24 h with destructive sampling occurring at 2, 4, 8, and 24 h. Membranes with *F. verticillioides* were transferred onto CZY plates without a bacterial ring culture to serve as negative controls. Fungal mycelium from a membrane was collected in labeled tubes from a 1.5 cm^2^ center circle marked with a sterile test tube cap, and the samples were flash-frozen in liquid nitrogen and stored at – 80°C until needed. The filter paper and the attached remaining mycelium were frozen in labeled aluminum foil and stored at – 80°C for backup material if needed.

### Sequencing

RNA was extracted from each mycelial sample in 1 mL lysis buffer and transferred to lysing matrix D tubes (MP Biomedicals, LLC, Santa Ana, CA, USA). Mycelia was homogenized with a FastPrep-24^™^5G instrument (MP Biomedicals) at 6 m/s with 2 pulses of 30 s and a 1 min intervening pause at room temperature. Total RNA of each sample was isolated with a PureLink RNA Mini Kit (Thermo Fisher Scientific Inc., MA, USA). An Agilent 2100 Bioanalyzer (Agilent Technologies, Palo Alto, CA, USA) was utilized to quality check RNA samples for degradation. An Illumina Truseq DNA LT sample prep kit (Illumina Inc., San Diego, CA, USA) was used to create sequencing libraries following the manufacturer’s protocol. For 2 and 4 h time points of all sample sequencing was done at the USDA/ARS facility in Stoneville, Mississippi USA. cDNA libraries were generated using Illumina TruSeq Stranded mRNA with Set A index adapters. Illumina library size validation was performed using the Agilent Tapestation 2200 High Sensitivity D1000 Assay (Part No. 5067–5584, Agilent Technologies, Santa Clara, CA, USA). Sequencing was performed on an Illumina HiSeq2500 DNA sequencer with SR Rapid v2 flowcell clustering kits (Product number GD-402-4002, Illumina, San Diego, CA, USA). Individual libraries were assayed for concentration by an Illumina library quantification kit (Product number KK4854, Kapa Biosystems, Inc, Wilmington, MA, USA) on a qPCR instrument (LightCycler 96, Roche Applied Science, Indianapolis, IN, USA). Each pool was clustered onboard an Illumina HiSeq2500 DNA sequencer with SR Rapid v2 flowcell clustering kits (Product number GD-402-4002, Illumina, San Diego, CA, USA). Single-end 50 bp sequencing was carried out with Rapid SBS v2 (Product number FC-402-4022, Illumina) reagents. Mycelial samples collected at 8 and 24 h were sent to Omega Bioservices (Norcross, Georgia, USA) for RNA extraction, cDNA library generation, and sequencing. RNA was extracted using Omega Bioteks’s E.Z.N.A.^®^ Total RNA Kit II (Product #: R6934-01). cDNA libraries were generated also using Illumina TruSeq Stranded mRNA (Product #: 20020595). Sequencing was performed on the Illumina HiSeq X Ten platform.

### RNA-Seq Analysis

#### Read Mapping and Quantification

Quality assessment of sequence reads was performed using FastQC (Andrews, 2010). Read adapters and low-quality sequences were removed using the default setting on Trimmoatic v0.39 (Bolger et al., 2014). The paired-end reads were aligned to the *F. verticillioides* M-3125 RefSeq genome from NCBI using HISAT2 v2.2.1 which utilizes bowtie2 (Pertea et al., 2016). SAMtools version 1.1.0 was implemented to sort the resulting files before HTSeq was run to obtain the read counts of all samples (Anders et al., 2014, Danecek et al., 2021). The GFF file used in this process was also downloaded from NCBI.

#### Identification of Differentially Expressed Genes (DEGs)

The tables of read counts generated by HTSeq were used as input for the R package, edgeR (Robinson et al., 2010). Using this package, samples of each time point were compared to the control to determine DEGs. It was at this time biological replicates were combined for the analysis. RPKM values were also determined using edgeR. Bash scripts were then utilized to parse the DEG and RPKM data.

#### Identifying Genes of Interest

To determine the biological significance of the DEGs, transcriptome data was compared to public databases. Functional annotation and predicted gene products of *F. verticillioides* were obtained from Uniprot, NCBI, and FungiDB. A list of transcription factors in *F. verticillioides* was obtained from the Fungal Transcription Factor Database (http://ftfd.snu.ac.kr/intro.php) (Park et al., 2008). While the fungal secretome database FunSecKB2 does not contain entries for *F. verticillioides*, data was obtained for *Fusarium oxysporum* genes (Meinken et al., 2014). Only *F. verticillioides* syntenic homologs of the *F. oxysporum* genes were identified from FungiDB and used in subsequent analysis. Analysis of secondary metabolite gene clusters was based on annotations done previously using multiple cluster prediction software (Gao et al., 2019). With these predictions, estimates of cluster sizes was based on the smallest range of overlapping genes and then used for identification. Also, numbering of the clusters was done for ease of identification which is reported in **Supplementary Table 2**. Information on clusters of characterized metabolites was incorporated based on established literature (Brown et al., 2012; Brown et al., 2015).

### RT-qPCR Verification

For RT-qPCR, RNA was prepared like above. A DNase treatment (Turbo DNA-free Kit, Thermo Fisher Scientific) was applied to samples prior to RT-qPCR. To confirm removal of DNA, standard Taq based PCR was performed with DNase treated RNA using RT-qPCR primers for β-tubulin (FVEG_04081). Presence of amplicons was assessed using gel electrophoresis. RT-qPCR reactions were carried out using a one-step RT-qPCR Kit (SuperScript III Platinum SYBR Green One-Step RT-qPCR Kit, Thermo Fisher Scientific) following the manufacturer’s protocol. Three technical replicates for each biological replicate were assayed with β-tubulin as the constitutive reference gene. Sequences of corresponding primer pairs for all genes used are listed in **Supplemental Table 3**. Expression was normalized to β-tubulin and the relative expression levels were calculated using the 2^−ΔΔCT^ method (Livak and Schmittgen, 2001).

### Deletion of a Strongly Induced Beta-Lactamase Gene, FVEG_13172

To construct *F. verticillioides* deletion mutants in gene FVEG_13172, the OSCAR deletion plasmid construction method (Gold et al., 2017) was carried out using primers FVEG_13172_OSCAR1-4, with sequences indicated in **Supplemental Table 3**. *Escherichia coli* (One Shot MAX Efficiency DH5αTM-T1R, Invitrogen, Carlsbad, CA, USA) was used as the recipient of the OSCAR deletion construct with selection on low sodium LB agar medium amended with 100 μg/mL spectinomycin at 37°C and DNA miniprepped from selected colonies cultured with shaking overnight at 37°C in/on low sodium (0.5 g/L) LB (Luria-Bertani) medium containing 100 μg/mL spectinomycin (Thermo Fisher Scientific, Waltham, MA, USA). After structural confirmation by restriction digests, several putative construct plasmids were sequenced (Integrated DNA Technologies, Coralville, Iowa, USA). A sequence confirmed OSCAR deletion plasmid was then introduced by transformation in *Agrobacterium tumefaciens* AGL-1 with selection on low sodium LB medium amended with 100 μg/mL spectinomycin at 27°C. A single AGL-1 transformant was streaked on LB-spectinomycin plates, incubated at 27°C for 48-72 h and used through co-cultivation with *F. verticillioides* M-3125 conidia as described (Paz et al., 2011; Gold et al., 2017). Fungal transformants were selected on potato dextrose agar (PDA; Neogen Food Safety, Lansing, MI, USA) plates containing 150 μg/ml hygromycin B (Invitrogen, Carlsbad, CA, USA) at 27°C. Gene deletion of FVEG_13172 was confirmed by PCR “anti-Southern” with a series of primer pairs (**Supplementary Table 3**, **Supplementary Figure 3**). The anti-Southern consists of four PCR potential amplicons for each transformant: *a)* confirmation of homologous recombination at the 5’ end with primers FVEG_13172_5’_Out and Hyg_Rev; *b)* absence of target ORF with primers FVEG_13172_ORF_For and FVEG_13172_ORF_Rev; *c)* introduction of hygromycin resistance gene with primers Hyg-ORF_For and Hyg-ORF_Rev; *d)* confirmation of homologous recombination at the 3’ end with primers Hyg_Fwd and FVEG_13172 3’_Out.

## Results

### *Fusarium verticillioides* Transcriptional Response to *Streptomyces* Isolates Varied Dramatically

**Figure 1:**
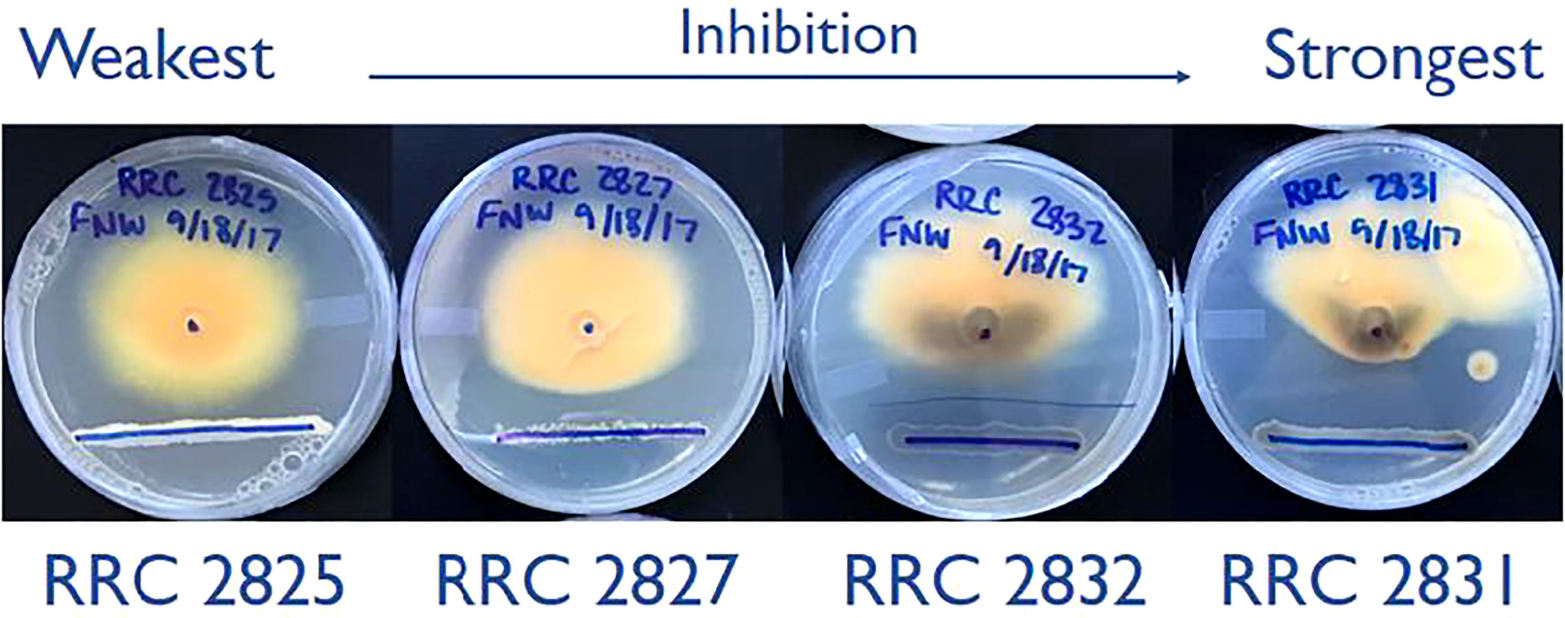
Dual-culture inhibition assays of the four *Streptomyces* strains used in this study. Strains spanning the full range of inhibition phenotypes were selected from an initial 9 tested. The lower blue sharpy lines indicate the locations of *Streptomyces* strain inoculation, grown for two days before inoculation of *F. verticillioides*. The central dot (25mm from the line) indicates where *F. verticillioides* spores were inoculated. Images captured 7 days after fungal spore inoculation.

**Figure 2:**
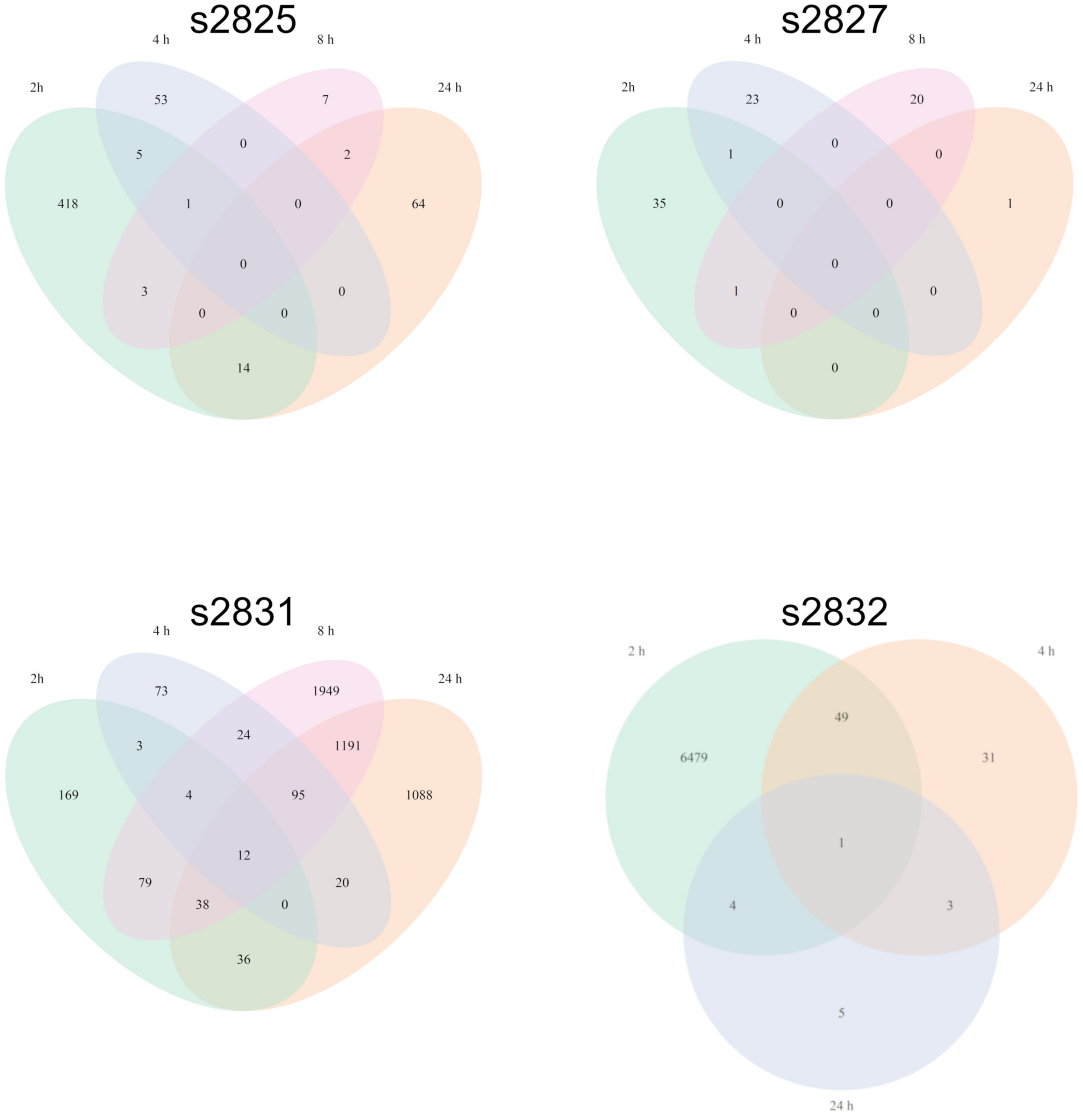
*Fusarium verticillioides* transcriptomic response to four *Streptomyces* strains. Venn diagrams representing the overlap of *F. verticillioides* DEGs over all timepoints for each *Streptomyces* strain dual-culture. The s2832 diagram does not include the 8 h timepoint since no statistically significant DEGs were detected.

Initially, *F. verticillioides* was exposed to nine environmental isolates of *Streptomyces via* confrontation assays (data not shown), and four of these (s2825, s2827, s2831, and s2832) demonstrated the range of responses (**Figure 1**) from no visible effect (s2825) to extreme inhibition (s2831). These four were chosen to assess distinct transcriptional responses in *F. verticillioides* to the spectrum of inhibitory effects, and as hypothesized, each of the four *Streptomyces* strains induced unique gene sets in *F. verticillioides* (**Figure 2**). Except for s2831, the 2 h time point produced the greatest transcriptomic temporal response in *F. verticillioides* of times assessed. After 2 h, *F. verticillioides* responses seemed to quench as it adapted to the presence of s2825, s2827, and s2832, with expression at later time points similar to the control. An example of this apparent adaptive quenching is the s2832 response at 8 h with no DEGs within our criteria even though there were over 6000 DEGs at the 2 h time point and 43 at 4 h. This rapid acclimatization was not witnessed with s2831, the strain with the greatest inhibition, as its response peaked the latest. At 2 h and 4 h, *F. verticillioides* vs s2831 had similar numbers of DEGs compared to s2825 interactions (<400). Later at 8 and 24 h, there was a large increase of *F. verticillioides* (> 1500) DEGs in response to s2831. The full results of the transcriptome analyses are documented in **Supplementary Table 4**.

### *Fusarium verticillioides* Responded Differently to Specific *Streptomyces* Isolates at Two Hours of Coculture

**Figure 3:**
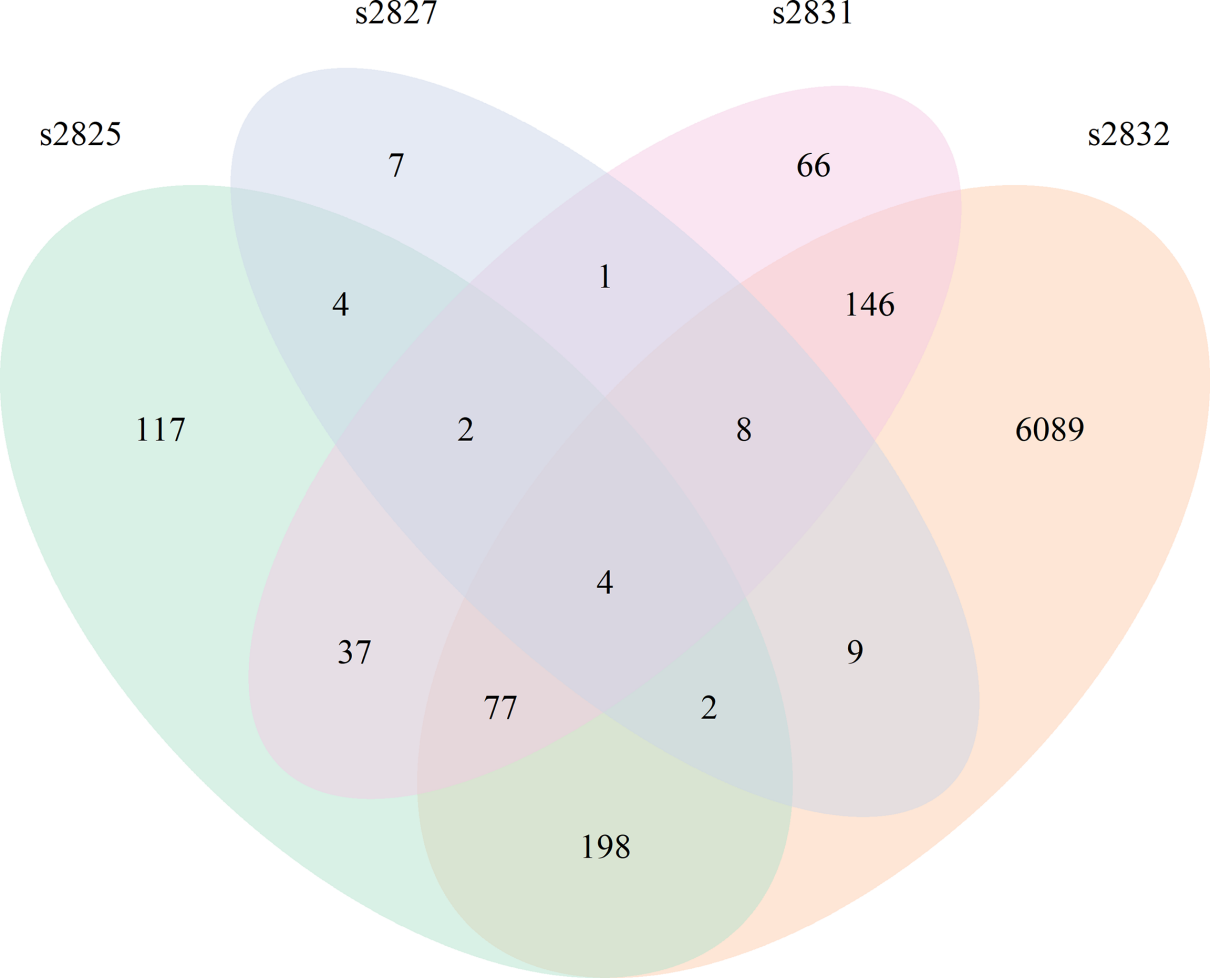
*Fusarium verticillioides* response to *Streptomyces* at 2 h. Venn diagram showing the overlap of DEGs from all 4 F*. verticillioides – Streptomyces* dual-cultures at 2 h.

All *Streptomyces* isolates induced a significant transcriptomic response by *F. verticillioides* with 2* h* of coculture (**Figure 3**). Comparison of the 2 h time points between isolates demonstrated slight overlap between differentially expressed *F. verticillioides* genes, but most were expressed in a strain-specific manner, particularly in the s2832 interaction with a much greater number of DEGs than the other samples. The number of genes uniquely expressed between strains varied greatly with 7 genes for s2827, 66 for s2831, 117 for s2825, and 6089 for the s2832. Among all interactions a common set of only four genes was induced in all interactions at 2 h. This set of 4 genes may represent an initial response of *F. verticillioides* to *Streptomyces* or antagonistic microbes more generally. Further, these DEGs are noted in interactions with strains like s2827 that cause minimal inhibition. Interestingly, s2827 induced very little transcriptome activity in comparison to the other strains (**Table 1**). One of the four common DEGs at the 2 h time point was FVEG_07988, a glutamate dehydrogenase and homolog of *Aspergillus nidulans gdhA*, a key enzyme in the assimilatory nitrogen pathway (Downes et al., 2013). *Streptomyces* s2832 suppressed activation of FVEG_07988 in contrast to the other isolates at 2 h. This may be an indication of different nutrients available to *F. verticillioides* created by *Streptomyces* competition where s2832 is actively competing for nitrogen with *F. verticillioides.* Two other predicted genes were FVEG_12768 and FVEG_13173 that encode an alcohol oxidase and a dimethylaniline monooxygenase, respectively. The last shared DEG is gene FVEG_11909, currently listed with no known function on FungiDB (https://fungidb.org/fungidb/app/record/gene/FVEG_11909) or NCBI (https://www.ncbi.nlm.nih.gov/gene/30069391). BLAST searches using the predicted amino acid sequence reveal no significant hits. However, using the nucleotide sequence identified potential homologs in other *Fusarium* spp. These putative FVEG_11909 homologs are frequently labled as predicted kinetochore complex Sim4 subunit Fta4. Closer examination of the sequence suggests that FungiDB mis-annotates the encoded protein in reading frame 2, while we detect the Sim4 subunit Fta4 amino acid homology in reading frame 1. The sequence annotated in FVER14953_11909 from a second *F. verticillioides* isolate, BRIP14953, deposited by D. Gardner in 2018 aligns well with other *Fusarium* orthologs, and we assume it is correct.

**Table 1:** Common differentially expressed genes at 2 h for all dual-cultures.

				Fold Change			
Gene	Gene_Name	Uniprot_Entry	Uniprot_Protein_Names	s2825	s2827	s2831	s2832
FVEG_07988	n/a	W7MAM7	Glutamate dehydrogenase	3.211414	2.257393	1.99652	-3.09575
FVEG_11909	n/a	W7MPX4	Uncharacterized protein	1.704116	2.387658	1.532398	-6.81215
**FVEG_12768**	**n/a**	**W7N4L9**	**Alcohol oxidase**	**3.869926**	**5.486566**	**5.113842**	**12.01406**
**FVEG_13173**	**n/a**	**W7NFR9**	**Uncharacterized protein**	**2.643388**	**2.968357**	**2.906794**	**11.13924**

Rows in bold are genes that show the same expression change in response to all Streptomyces strains.

n/a, not available.

**Figure 4:**
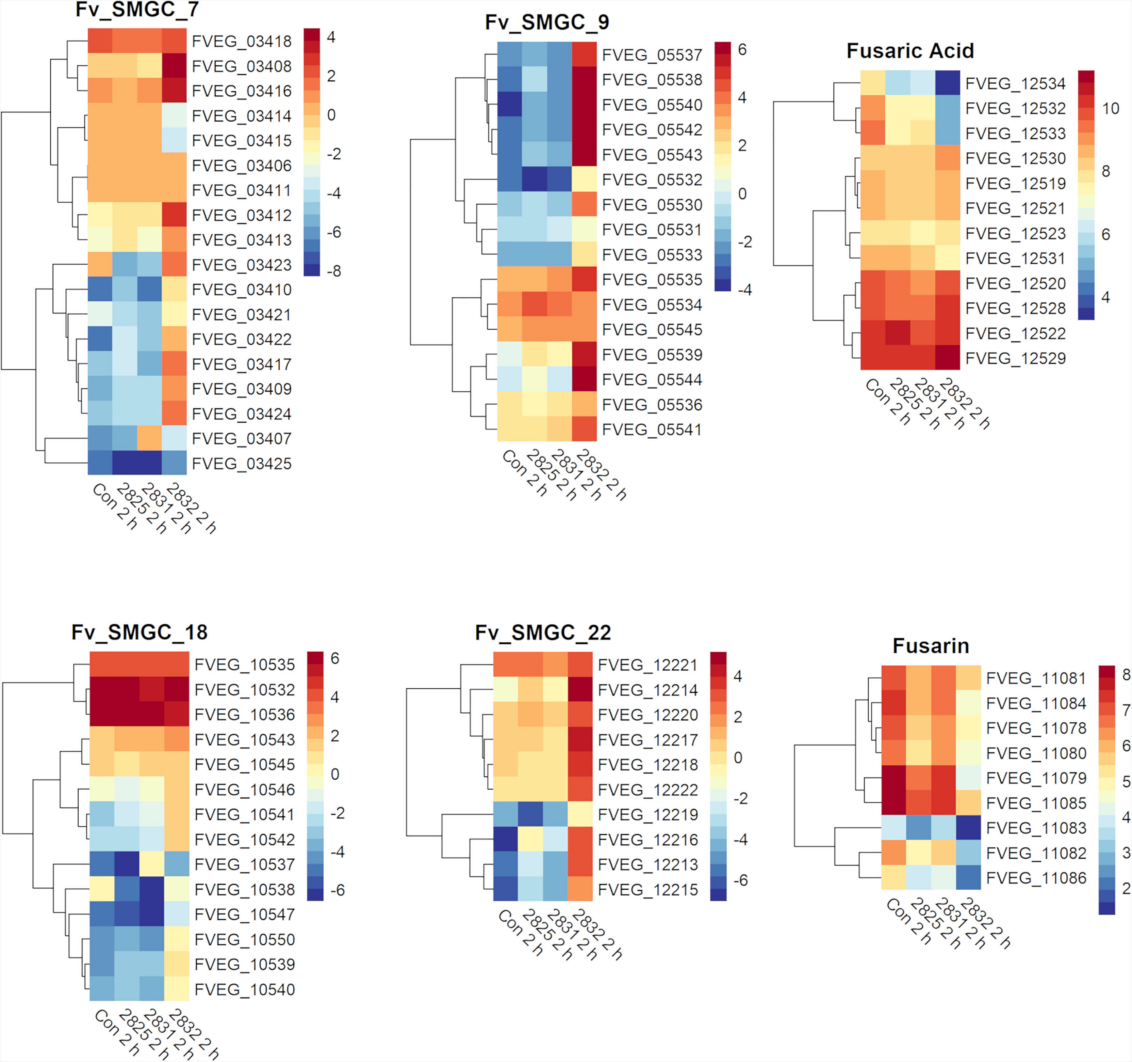
SMGCs induced at 2 h after exposure to *Streptomyces.* Heat maps of *F. verticillioides* RPKM values of fusaric acid and fusarin clusters and SMGC 7, 9 18, and 22 at 2 h. RPKM values were logarithmically transformation before being visualized by R package “pheatmaps”. The scale bars represent these logarithmic values. The data are presented for the 2 h exposures with *Streptomyces* strains s2825, s2831, and s2832. The s2827 interaction is not reflected in this figure as no genes (except FVEG_03697) were differentially expressed with respect to secondary metabolism gene clusters.

After 2 h exposure, the transcriptional response to s2832 included large alterations in *F. verticillioides* secondary metabolite biosynthetic gene clusters (**Figure 4**). Secondary metabolite gene clusters (SMGCs) 7, 9, 18, and 22 were all upregulated when *F. verticillioides* was challenged with s2832. While the metabolites produced by these clusters are not known, clusters for production of fusaric acid and fusarin were also affected by s2832. In comparison to the control, s2832 samples showed reduced expression of the fusarin cluster and a portion of the fusaric acid cluster, including both transcription factors (FVEG_12532 and FVEG_12534). With s2825 and s2831, some genes in the fusarin cluster also showed reduced expression but not to the same degree as with s2832. *Streptomyces* strain s2827 caused significant differential expression of only one SMGC gene, FVEG_03697, and thus it is not reflected in **Figure 4**.

Both fusarin and fusaric acid are considered mycotoxins, with fusarin demonstrating mutagenic and carcinogenic attributes, while fusaric acid may be involved in inhibition of ATP synthesis and chelation of metal ions (Lu et al., 1989; Li, 1992; Ruiz et al., 2015, Lopez-Diaz et al., 2017). Multiple studies have demonstrated harmful effects of fusarin and fusaric acid on bacteria (May et al., 2000; Ruiz et al., 2015, Tung et al., 2017). However, for *Streptomyces*, no direct studies to our knowledge have been found involving these mycotoxins. However, on the UniProt database, there are multiple strains of *Streptomyces* with a computationally predicted fusaric acid resistance protein (UniProt Accession #:A0A5B7V4R7) which indicates a possible adaption to this *Fusarium* mycotoxin from this species group.

### Strain s2831 Induced a Prolonged Transcriptional Response in *F. verticillioides*

In the current study, three of the *Streptomyces* strains induced an intial gene expression response that peaked early in *F. verticillioides*, then quenched. This was not the case with s2831 where *F. verticillioides* differential expression peaked at 8 h but remained high even at 24 h. Despite over 2,000 DEGs being identified at the later time points, only a small number of them were induced at all time points (**Table 2**). A subset of these genes encodes proteins associated with stress response such as FVEG_00720 (heat shock-like protein), FVEG_07470 (chaperone protein HptG), and FVEG_11780 (small heat shock protein). DEGs induced by s2831 were assessed for transcription factors, secretory genes, and those in SMGCs (**Supplementary Figure 4**). While genes were uniquely expressed in each category, none were identified across all timepoints. A cause for this extended and varied response might be metabolic changes in the s2831 strain after encountering *F. verticillioides*. This scenario would account for the weaker early response seen at 2 and 4 h as s2831 may produce active defense compounds once *F. verticillioides* is encountered.

**Table 2:** Common differentially expressed genes for s2831 dual-cultures across all time points.

				Log2 Fold Change			
Gene	Gene_Name	Uniprot_Entry	Uniprot_Protein_Names	2h	4h	8h	24h
FVEG_00720	n/a	W7LBG6	Hsp70-like protein	1.26699	1.888862	2.61836	2.08853
**FVEG_04564**	**n/a**	**W7LUG6**	**AAA domain-containing protein**	**1.10331**	**4.225458**	**10.5094**	**6.92737**
**FVEG_06219**	**n/a**	**W7M366**	**Uncharacterized protein**	**2.41461**	**2.391367**	**5.39618**	**4.40134**
FVEG_07114	n/a	W7M703	APH domain-containing protein	1.37977	2.857786	5.28555	5.19558
FVEG_07470	n/a	W7MRX2	Molecular chaperone HtpG	1.73754	1.941439	2.94037	2.41759
**FVEG_09349**	**n/a**	**W7MGK6**	**D-aminopeptidase**	**1.78611**	**3.354081**	**5.98613**	**4.40856**
**FVEG_11780**	**n/a**	**W7NA05**	**SHSP domain-containing protein**	**1.93652**	**5.165589**	**8.42438**	**6.86376**
**FVEG_12080**	**n/a**	**W7MRJ5**	**Sarcosine oxidase**	**1.88064**	**7.49095**	**10.7756**	**9.24146**
FVEG_12613	n/a	W7NDM5	ThuA domain-containing protein	1.51846	3.150384	4.40004	4.57989
**FVEG_12951**	**n/a**	**W7N5C1**	**EHN domain-containing protein**	**4.02711**	**5.53025**	**10.6973**	**6.17062**
**FVEG_13374**	**n/a**	**W7MV20**	**Uncharacterized protein**	**1.46095**	**3.935986**	**4.71681**	**3.66693**
**FVEG_17540**	**n/a**	**W7N749**	**HET domain-containing protein**	**2.89308**	**3.432834**	**7.59493**	**5.88926**

Genes in bold are genes with expression profiles that mimics the response to Streptomyces with peak response being at 8 hours before decreasing.

n/a, not available.

### Interaction With s2831 Caused Early and Strong Activation of Predicted *F. verticillioides* Secondary Metabolite Gene Clusters

**Figure 5:**
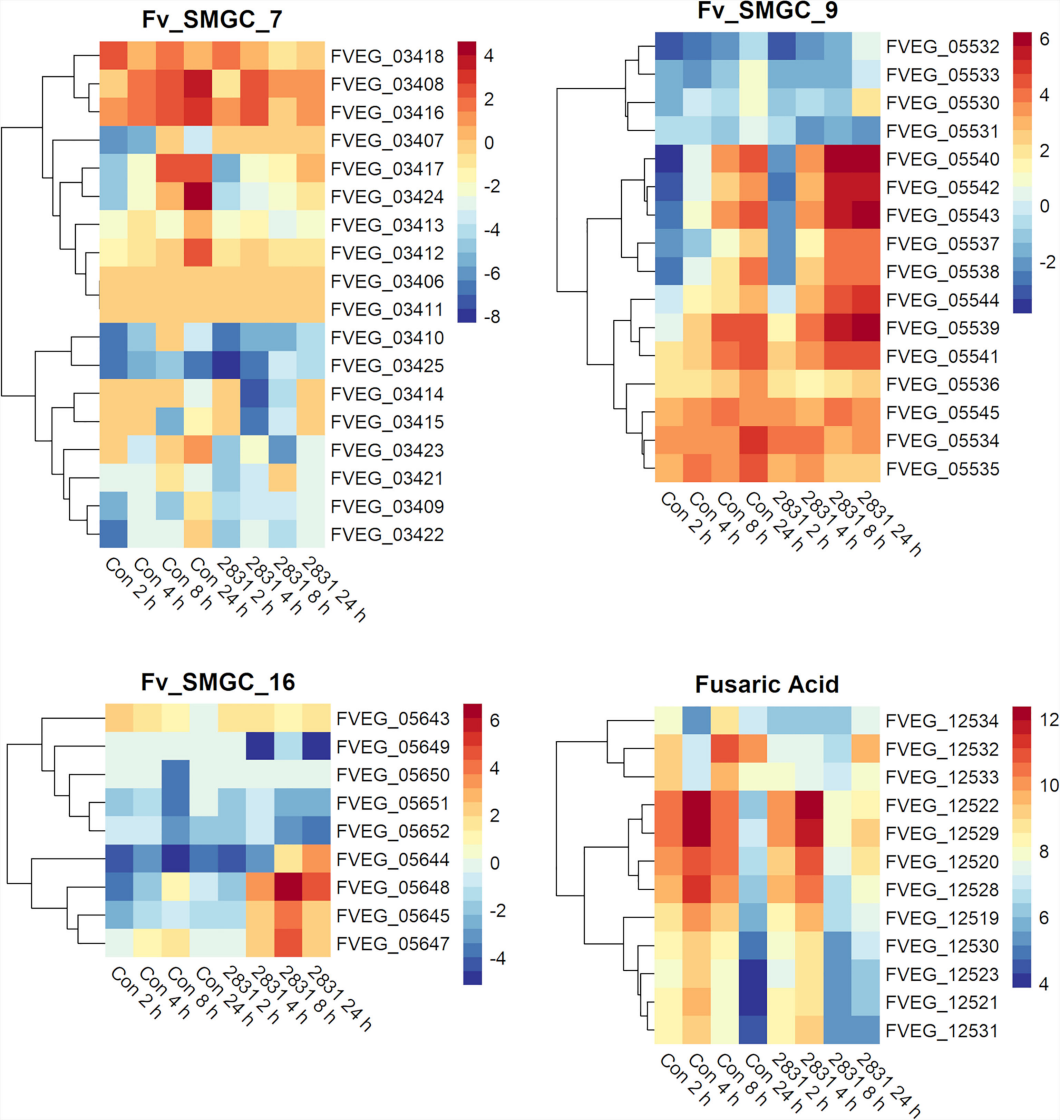
SMGCs in response to dual-culture with *Streptomyces* strain s2831. Heat maps of RPKM values of SMGC 7, 9, 16, and fusaric acid clusters showing expression by *F. verticillioides* in response to s2831. RPKM values underwent a logarithmic transformation to be visualized by R package “pheatmaps”. The scale bars represent these transformed values.

Several predicted *F. verticillioides* gene clusters were affected by s2831 (**Figure 5**). Clusters 9 and 16 showed increased expression with exposure to s2831. Over one third of Cluster 9 (FVEG_05539 to FVEG_05544) was transcriptionally activated earlier (4 h vs 8 h) and more strongly when *F. verticillioides* was exposed to s2831 compared to monoculture (**Figure 5**). This trend continued at 8 and 24 h with strong induction of the cluster. Cluster 16 appears to be a cryptic cluster as genes of this cluster are not constitutively expressed but activate with *F. verticillioides* response to s2831 (**Figure 5**). Additionally, cluster 7 and the fusaric acid gene cluster were suppressed in comparison to the control (**Figure 5**). For cluster 7, the upper half of the heat map shows more expression than the lower half which is weakly induced even in the control. This is true for both control and s2831 samples, but samples confronted with *Streptomyces* are more weakly induced or further suppressed compared to the control. At the fusaric acid cluster at 8 h, the non-ribosomal peptide synthetase (FVEG_12530) and both transcription factors (FVEG_12532 and FVEG_12534) demonstrated reduced expression.

### *F. verticillioides* Transcriptional Response to Inhibitory Isolates of *Streptomyces*

Not surprisingly the strains with the largest impact on *F. verticillioides* growth, s2831 and s2832, also caused a broad transcriptomic response. This response suggests that *F. verticillioides* combats and adapts to individual *Streptomyces* strains in specific ways. A comparison was made of DEGs from the highly expressed samples of s2831 2h and s2832 8h and 24 h treatments (**Figure 5**). Additionally, genes from each group were selected and expression trends confirmed by qRT-PCR (**Supplementary Table 5**). Among these three samples, 671 DEGs were shared, which may indicate a conserved genetic response to strongly inhibitory strains of *Streptomyces* by *F. verticillioides.* Of the shared DEGs, over 80% have no annotated function. Despite many having no predicted function, several annotated DEGs have been identified that may have a role in the response of *F. verticillioides.* Several stress-related genes that may be part of this response are chaperone proteins BCS1 (FVEG_03843) and DnaJ (FVEG_06380) as well as a heat shock-like protein (FVEG_06113). Multiple transcriptional elements were also found in both transcriptomes including FVEG_01821 (transcription initiation factor TFIIH subunit 4), FVEG_02570 (DNA-directed RNA polymerase III subunit RPC1), FVEG_03031 (translation initiation factor eIF-2B subunit beta), FVEG_10353 (DNA-directed RNA polymerase III subunit RPC7), and FVEG_11419 (pre-mRNA-splicing factor ATP-dependent RNA helicase *prp43*). Strangely though these elements were repressed by s2832 while induced by s2831, with the exception of FVEG_03843 which was induced by both strains.

**Figure 6:**
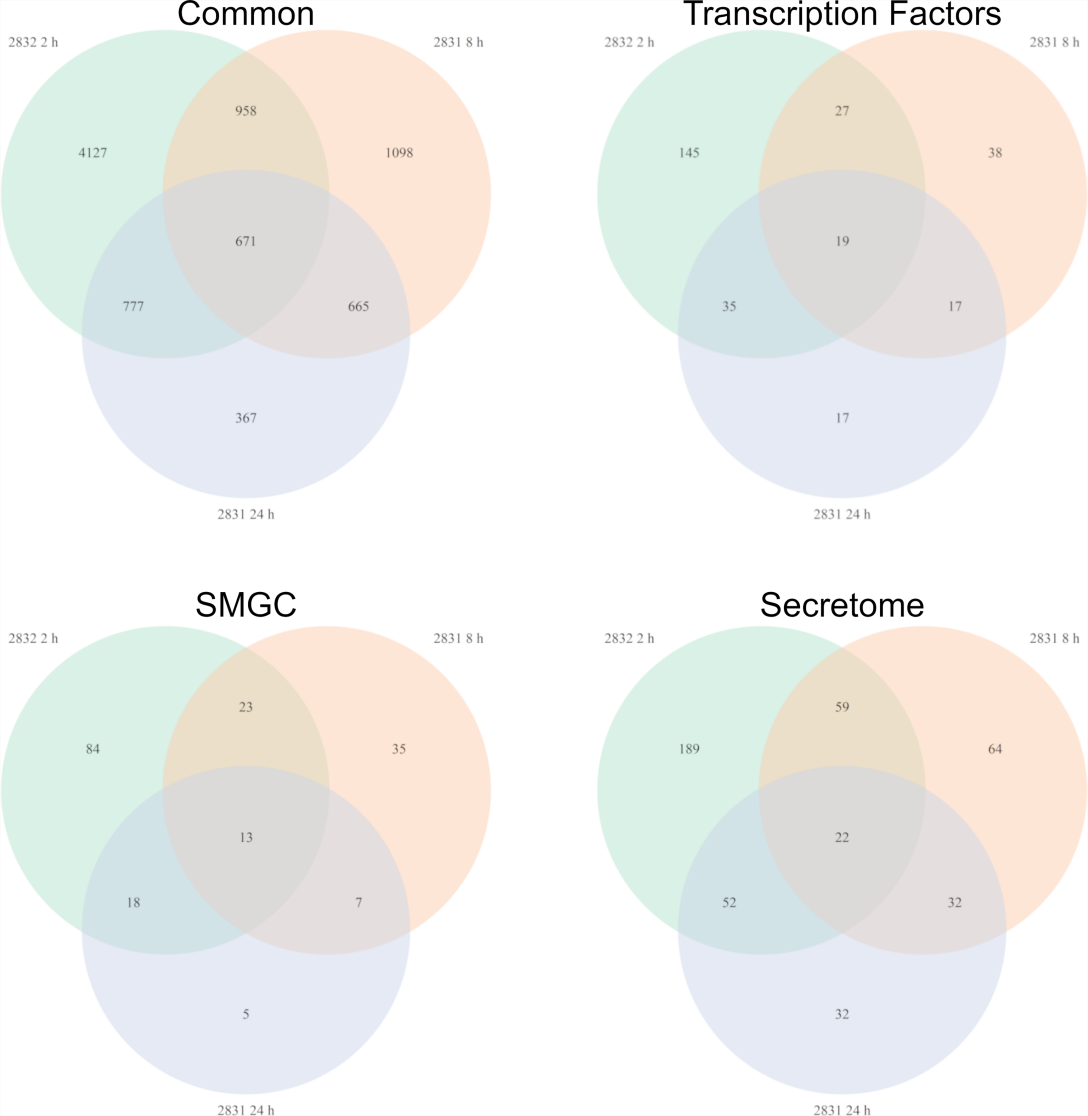
*Fusarium verticillioides* genes expressed in response to *Streptomyces* strain s2831 that cause strong inhibition. The number of DEGs was highest at 2 h for the *Fv-*s2832 interaction and 8 and 24 h with strain s2831. Comparisons to find similarly expressed genes were made and visualized by Venn Diagrams. Additional comparisons were made to parse gene encoding for transcription factors, secondary metabolite gene clusters, and secretome encoding genes.

To glean more information from the common gene responses, their expression profiles from databases were reviewed (**Figure 6**). Out of the 13 transcription factors that were conserved, all but two showed different trends in expression between the treatment strains. The two transcription factors with consistent expression patterns were FVEG_12686 and FVEG_12938. FVEG_12686 is uncharacterized while syntenic homologs of FVEG_12938 are related to TRI-15, a transcription factor regulating trichothecene mycotoxin production (Alexander et al., 2004). FVEG_08110, a homolog of the Pro-1 virulence factor in *Fusarium graminearum* which positively regulates virulence in wheat (Son et al., 2011), was also differentially expressed, but at the 2 h time point it was repressed by s2831 while induced by s2832. Concerning SMGCs only a small part of cluster 7, including a gene encoding a PKS (polyketide synthase) domain-containing product (FVEG_03423), was significantly differentially expressed in all three highly expressed groups. Between strains, part of cluster 7 was induced by s2831 while suppressed with s2832. In contrast to the transcription factors and SMGCs, no clear trend is demonstrated among the conserved secretome components.

Examination of the exposure responses of *F. verticillioides* to all four strains, identified a few genes that stand out for high expression when the fungus is suppressed by *Streptomyces.* Both FVEG_11780 and FVEG_12951 are active in the response outside of the high expression time points when *F. verticillioides* is exposed to s2831. For s2831, both genes increase in expression before peaking at 8 h. A similar situation was observed in confrontation with s2832, in that case expression of these two genes peaked at 2 h before subsiding. These two genes are predicted to encode a small heat shock protein (FVEG_11780) and an epoxide hydrolase (FVEG_12951) and are likely part of a self-protection mechanism in response to inhibition/toxicity.

### Deletion of Beta-Lactamase FVEG_13172 Increases Response of *F. verticillioides* to *Streptomyces* Xenobiotics

**Figure 7:**
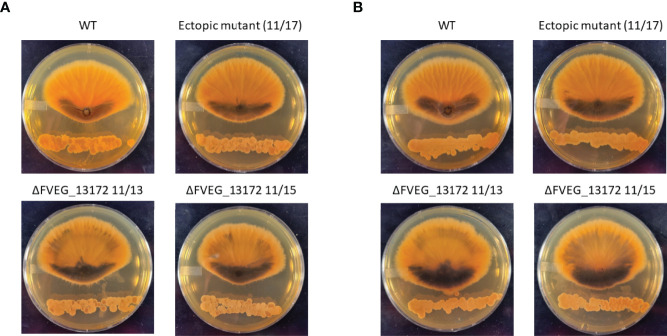
Effect of ΔFVEG_13172 on inhibition caused by dual culture with *Streptomyces* strain s2831 or s2832. **(A)** Interactions of *F verticillioides* with s2831. **(B)** Interactions of *F verticillioides* with s2831*Streptomyces* strain s2381 and s2832 were inoculated and grown for two days before inoculation with *F verticillioides* 25* mm* from initial *Streptomyces* inoculation. The M3125 WT strain, two ΔFVEG_13172 strains (11/13 & 11/15) and one ectopic transformant (11/17) were used in this experiment. Images captured 7 days after fungal spore inoculation.

In previous research we identified 46 genes encoding proteins annotated as β-lactamases in *F. verticillioides* and also that β-lactamases are generally very common in soilborne fungi suggesting that hydrolysis of lactam moiety containing compounds is a significant environmental defense mechanism for these organisms (Gao et al., 2017; Gao et al., 2020). In the current work we found the lactamase encoding gene FVEG_13172 to be a DEG in both the 2h s2832 (8-fold increase) and 24 h s2831 (5-fold decrease) dual-cultures. To test the role of this gene in responding to the various *Streptomyces* strains multiple deletant strains of this gene were created in *F. verticillioides* (**Supplementary Figure 3**). The initial experiment identifying inhibitory *Streptomyces* strains was repeated with s2831 and s2832 in confrontation with the FVEG_13172 mutant strains (**Figure 7**). Despite the difference in transcriptomic response at early time points, deletion of FVEG_13172 created similar phenotypes in longer term exposure to both repressive *Streptomyces* strains. In confrontation cultures with the mutants, the production of a black pigment is more pronounced on the interacting side of the fungal colony and also accumulates elsewhere in the colony. It can be inferred that with the loss of this lactamase encoding gene and resultant phenotype, at least one of the compounds inhibiting *F. verticillioides* produced by s2831 and/or s2832 is likely to be a lactam molecule. Additionally, it would seem *F. verticillioides* has multiple layers of response/defense to this lactam. For example, if FVEG_13172 was the sole response gene its removal would cause a more severe phenotype, including possibly being completely unable to grow next to the *Streptomyces* strains. A reported example that supports this concept involves *F. verticillioides* metabolism of the plant antimicrobial γ-lactam, 2-benzoxazolinone (BOA). Deletion of the lactamase MBL1 encoding gene causes *F. verticillioides* to be incapable of metabolizing BOA and thus unable to grow on media containing the compound (Glenn et al., 2016).

## Discussion

In the soil environment, *F. verticillioides* interacts with a wide variety of microbes including *Streptomyces* bacteria. While an initial hypothesis might have been that *F. verticillioides* has a common response to different inhibitory strains of *Streptomyces*, we show here this may only be the case initially. The onset of these responses varies temporally amongst the strains assayed from a very quick induction (2 h for s2831 exposure) to a response that crescendos hours after exposure (8 h in response to s2832). Only four genes were differentially expressed in common among the four strains at the 2 h time point. None of these genes have been investigated in *F. verticillioides*. For one gene, FVEG_07988, a homolog characterized in *A. nidulans* known as *gdhA*, was shown to be involved in the nitrogen assimilation pathway (Downes et al., 2013).

In response to the two strongly inhibitory strains of *Streptomyces*, s2831 & s2832, the expression of the fusaric acid biosynthetic gene cluster was affected. The sensitivity to this mycotoxin may have led to selection for metabolites produced by certain *Streptomyces* strains able to inhibit fusaric acid production. A fungal-fungal example of this type of metabolite cross-talk involving fumonisin and pyrrocidine was recently reported by our group (Gao et al., 2020). Pyrrocidine is produced by *Sarocladium zeae*, a corn endophyte that commonly encounters *F. verticillioides* (Wicklow et al., 2005). Pyrrocidine was shown to suppress fumonisin production in *F. verticillioides* at well below growth inhibitory concentrations (Gao et al., 2020). Suppression of the fusaric acid cluster in response to the two strongly inhibitory *Streptomyces* strains used in this study suggests that it might be a problematic compound for *Streptomyces* as well. This suggests that certain *Streptomyces* strains are likely more successful growing in competition with *F. verticillioides* due to this capacity than are others. While one toxin may be suppressed, *F. verticillioides* has multiple lines of defense against competing/attacking microbes. For fungi in general the main line of defense is chemical (Künzler 2018). Some of these defense chemicals constitutively produced, including fusaric acid which can be produced in monoculture, while production of some other defense compounds are induced in response to antagonism (Brown et al., 2012, Künzler 2018). We observed examples of antagonist induction in the dual culture using s2831, where both cluster 9 and 16 initiate induction at the 8 h time point and continue into the 24 h time point. Neither cluster 9 nor 16 was induced at any time point by the minimal inhibitory strains, s2825 and s2827. Thus, induction of cluster 9 and 16 may be considered a potential defense response to the stress caused by the inhibitory compounds produced by strain s2831.

With only four *F. verticillioides* genes commonly induced by all tested strains it is not possible to assign a primary *Streptomyces* induced signal or regulatory element. However, analysis of the two most inhibitory strains provided important clues regarding a shared response to different inhibitory strains by *F. verticillioides.* In response to the inhibition from the *Streptomyces* metabolites, stress response protein encoding genes like chaperones and heat shock proteins were induced. In *Saccharomyces cerevisiae*, it was determined that suppression of RNA polymerase III by desumoylation of its subunits contributed to regulating the stress response to multiple abiotic stressers like oxidative stress although the specificity of the response was unclear (Nguéa P et al., 2019). Suppression of RNA polymerase III was also encountered in another metabolic stress-based study (Leśniewska et al., 2019). Two homologs of units of RNA polymerase III, FVEG_02570 (DNA-directed RNA polymerase III subunit RPC1) and FVEG_10353 (DNA-directed RNA polymerase III subunit RPC7), were found to be DEGs at 2 h and 8 h for s2832 and s2831 co-inoculations respectively. Based on the temporal delay as well as difference in expression profile (induced vs suppressed) this may be the case between the different transcript profiles as these subunits show opposite expression profiles with s2831 and s2832. This lends additional credence to the idea that s2831 and s2832 are producing different inhibitory compounds that trigger unique and sometimes opposing transcriptional responses in *F. verticilliodes*.

*Fusarium verticillioides* posesses forty-six genes described as encoding beta lacatamases (Gao et al., 2017). The lactamase FVEG_13172 was highly induced by the two most inhibitory strains of *Streptomyces*. Deletion of this gene rendered *F. verticilliodes* mutants visibly more responsive to the strongly inhibitorory strains. This result suggests that fungal defense against antagonisitic *Streptomyces* involves numerous strategies including likely resistance to specific lactam molecules.

In summary this study demonstrates that some, but certainly not all, *Streptomyces* strains may have potential for biological control of *F. verticillioides*. Exploration of agriculturally important *Fusarium* host crop environments may allow identification of even more competitive strains of *Streptomyces* that may have better biocontrol potential.

## Data Availability Statement

The datasets presented in this study can be found in on-line repositories. The names of the repository/repositories and accession number(s) can be found on NCBI using the BioProject ID: PRJNA764855.

## Author Contributions

Conceptualization, SG and AG. Methodology, SG, TS, and MN. Validation, TS. Formal analysis, TS and FW. Investigation, TS, FW, MN, and SG. Resources, SG and AG. Data curation, TS. Writing – original draft preparation, TS. Writing – review and editing, TS, SG, and AG. Supervision, TS and SG. Project administration, SG. Funding acquisition, SG and AG. All authors contributed to the article and approved the submitted version.

## Funding

Funding for this project comes from USDA/ARS Project Number: 6040-42000-045-00-D.

## Conflict of Interest

The authors declare that the research was conducted in the absence of any commercial or financial relationships that could be construed as a potential conflict of interest.

## Publisher’s Note

All claims expressed in this article are solely those of the authors and do not necessarily represent those of their affiliated organizations, or those of the publisher, the editors and the reviewers. Any product that may be evaluated in this article, or claim that may be made by its manufacturer, is not guaranteed or endorsed by the publisher.

## Supplementary Material

Supplementary Table 1 Strains used in this study.

Supplementary Table 2 Numbered list of secondary metabolite gene clusters predicted in *F. verticillioides.*

Supplementary Table 3 List of primers used in this study.

Supplementary Table 4 Complete analysis of transcriptomic data.

Supplementary Table 5 Comparison of expression of selected genes in RNA-Seq and RT-qPCR.

Supplementary Figure 1
*Fusarium verticillioides-Streptomyces* dual-culture experimental design. **(A)** Growth of *Streptomyces* and *F. verticillioides* cultures after 48 h of solo growth. **(B)** *F. verticillioides* grown filter, after 2 days of growth, placed (without direct contact) within the ring of *Streptomyces* strain growth. **(C)** Mycelial sample collection.

Supplementary Figure 2 RT-qPCR verification of select DEGs. To verify observed RNA-Seq differential expression, RT-qPCR was performed on select genes. **(A)**, **(B)**, and **(C)**, correspond to genes from the s2832 2 h, s2831 8 h, and s2831 24 h samples, respectively. Note that scales vary greatly, adjusted to show expression of each gene normalizing the control treatment as a value of 1.

Supplementary Figure 3 Design and confirmation of gene deletion of induced beta-lactamase FVEG_13172. **(A)** Deletion strategy for FVEG_13172 with color coded primer pairs used in production of OSCAR deletion plasmid and confirmation of gene deletion. **(B, C, D, E)** Agarose gel electrophoresis “Anti-Southern” PCR deletion mutant confirmation for *Fusarium verticillioides* ΔFVEG_13172 (11/13-11/15) and ectopic transformants (11/17 & 11/18). A 1kb ladder was used in all gels. **(B)** Confirmation of homologous recombination at the 5’ end of the gene with primers FVEG_13172_5’_Out and Hyg_Rev. **(C)** Presence/absence of FVEG_13172 open reading frame with primers FVEG_13172_ORF_For and FVEG_13172_ORF_Rev. **(D)** hygromycin resistance gene in transformants with primers Hyg-ORF_For and Hyg-ORF_Rev. **(E)** confirmation of homologous recombination at the 3’ end with primers Hyg_Fwd and FVEG_13172 3’ Out.

Supplementary Figure 4 Differential expression time course of *F. verticillioides* gene encoding for transcription factors, secondary metabolism clusters, and secretome in response to dual-culture with *Streptomyces* strain s2831. A list of identified and predicted genes corresponding to transcription factors, secondary metabolism, and secretome was compared to DEGs from the s2831 dataset and represented as Venn diagrams.
