# Supplementary figures and images for: Transcriptomic Response of Fusarium verticillioides to Variably Inhibitory Environmental Isolates of Streptomyces
Source: Front Fungal Biol. 2022 Jul 28;3:894590. doi: 10.3389/ffunb.2022.894590 (PMC10512263; doi:10.3389/ffunb.2022.894590)

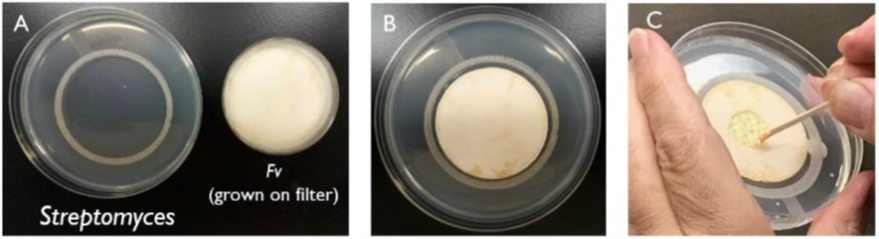

Supplement: Supplementary Figure 1 — Fusarium verticillioides-Streptomyces dual-culture experimental design. (A) Growth of Streptomyces and F. verticillioides cultures after 48 h of solo growth. (B) F. verticillioides grown filter, after 2 days of growth, placed (without direct contact) within the ring of Streptomyces strain growth. (C) Mycelial sample collection. [file Image_1.tif]

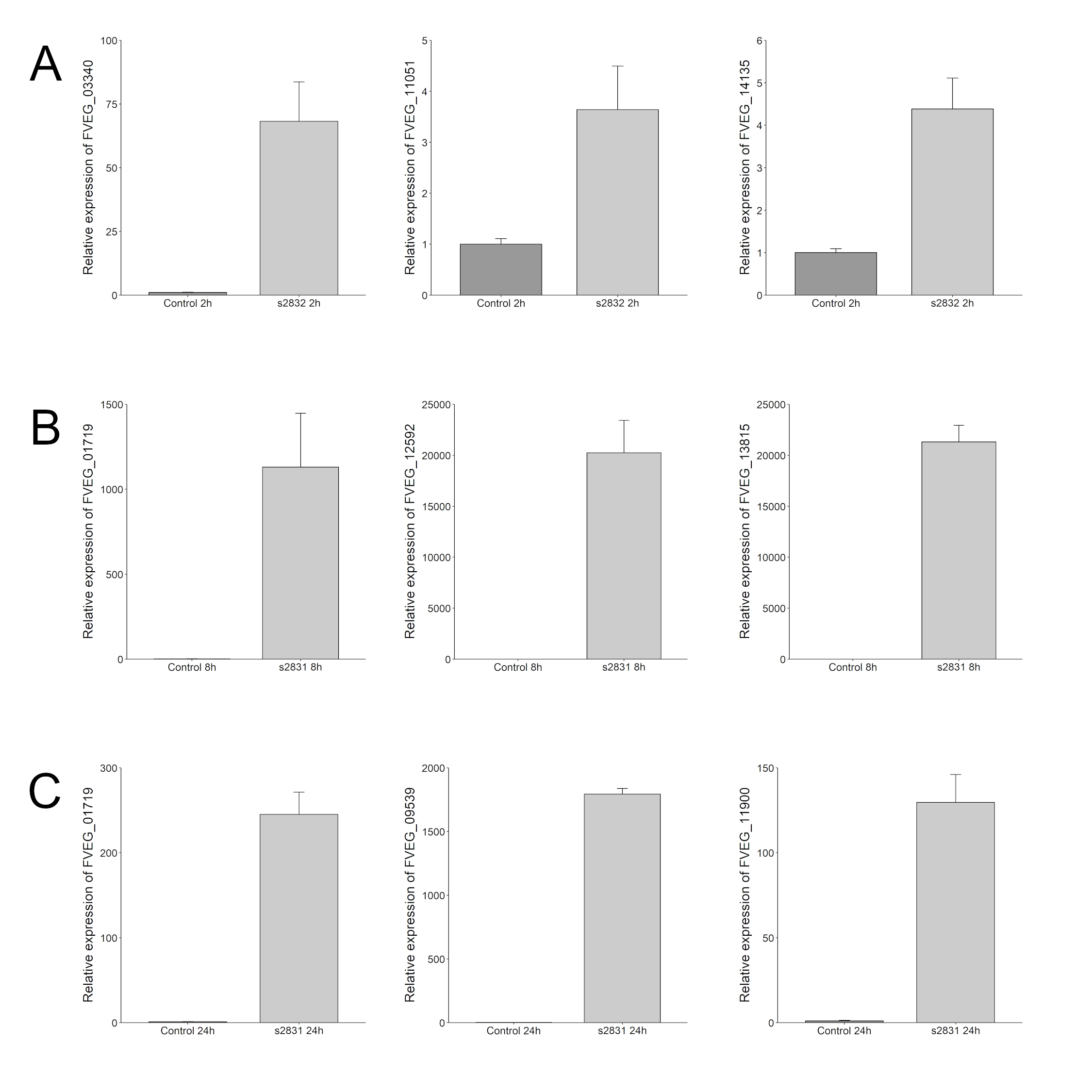

Supplement: Supplementary Figure 2 — RT-qPCR verification of select DEGs. To verify observed RNA-Seq differential expression, RT-qPCR was performed on select genes. (A), (B), and (C), correspond to genes from the s2832 2 h, s2831 8 h, and s2831 24 h samples, respectively. Note that scales vary greatly, adjusted to show expression of each gene normalizing the control treatment as a value of 1. [file Image_2.tiff]

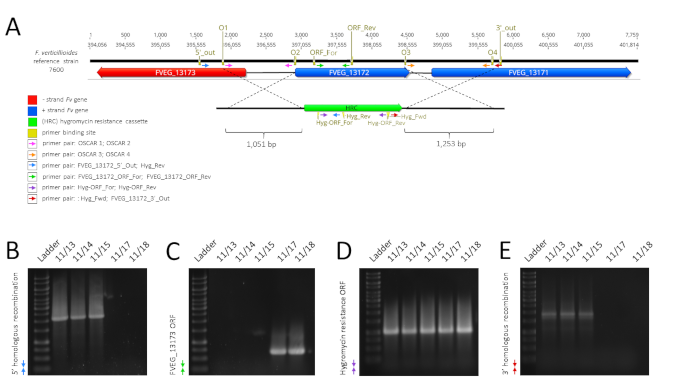

Supplement: Supplementary Figure 3 — Design and confirmation of gene deletion of induced beta-lactamase FVEG_13172. (A) Deletion strategy for FVEG_13172 with color coded primer pairs used in production of OSCAR deletion plasmid and confirmation of gene deletion. (B, C, D, E) Agarose gel electrophoresis “Anti-Southern” PCR deletion mutant confirmation for Fusarium verticillioides ΔFVEG_13172 (11/13-11/15) and ectopic transformants (11/17 & 11/18). A 1kb ladder was used in all gels. (B) Confirmation of homologous recombination at the 5’ end of the gene with primers FVEG_13172_5’_Out and Hyg_Rev. (C) Presence/absence of FVEG_13172 open reading frame with primers FVEG_13172_ORF_For and FVEG_13172_ORF_Rev. (D) hygromycin resistance gene in transformants with primers Hyg-ORF_For and Hyg-ORF_Rev. (E) confirmation of homologous recombination at the 3’ end with primers Hyg_Fwd and FVEG_13172 3’ Out. [file Image_3.tiff]

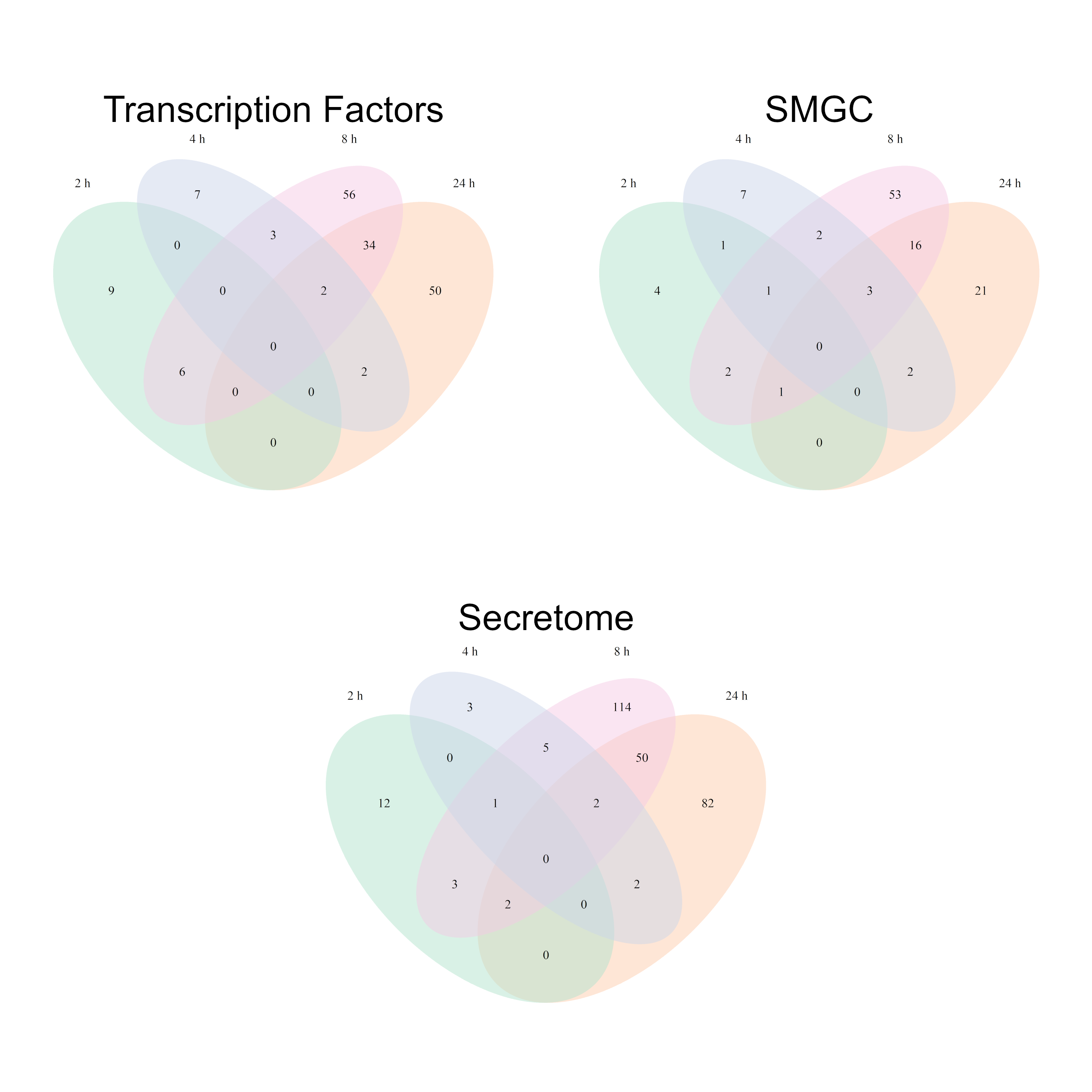

Supplement: Supplementary Figure 4 — Differential expression time course of F. verticillioides gene encoding for transcription factors, secondary metabolism clusters, and secretome in response to dual-culture with Streptomyces strain s2831. A list of identified and predicted genes corresponding to transcription factors, secondary metabolism, and secretome was compared to DEGs from the s2831 dataset and represented as Venn diagrams. [file Image_4.tiff]
